# Lumbar puncture safety and tolerability in premanifest and manifest Huntington’s disease: a multi-analysis cross-sectional study

**DOI:** 10.1038/s41598-022-21934-6

**Published:** 2022-11-01

**Authors:** Yara Refaat Hassan, Filipe Brogueira Rodrigues, Paul Zeun, Lauren M. Byrne, Carlos Estevez-Fraga, Rosanna Tortelli, Rachael I. Scahill, Edward J. Wild, Sarah J. Tabrizi

**Affiliations:** UCL Huntington’s Disease Centre, UCL Queen Square Institute of Neurology, 10-12 Russell Square House, London, WC1B 5EH UK

**Keywords:** Neurodegenerative diseases, Huntington's disease

## Abstract

Lumbar puncture (LP) has become increasingly common for people with Huntington’s disease (HD) both to administer intrathecal investigational medicinal products and to collect cerebrospinal fluid to develop biological markers to track disease stage and progression. We aimed to investigate the safety profile of LP in people with HD, building on a recently published work by increasing the sample size and more specifically, increasing the representation of the premanifest population and healthy controls. We conducted a multi-study cross-sectional analysis including eligible participants from the HDClarity (304 Huntington's disease gene expansion carriers and 91 controls) and HD-YAS studies (54 premanifest and 48 controls), enrolled between February 2016 and September 2019. We investigated the odds of any adverse events, headaches, and back pain independently. Intergroup comparisons and adjusted event odds were derived using hierarchical logistic regressions. A total of 669 LP procedures involving 497 participants were included in this analysis. There were 184 (27.5%) LP procedures associated with one or more adverse events. The two most common adverse events were: post LP headache and back pain. Younger age and female gender were found to be associated with a higher risk of developing adverse events. There was no difference in the rate of adverse events between the disease subgroups after adjusting for covariates such as age and gender. Our results suggest that the LP is safe and tolerable in premanifest and manifest HD subjects, providing useful reassurance about the procedure to the HD community.

## Introduction

Huntington’s disease (HD) is an autosomal dominant, progressive neurodegenerative disease resulting from CAG trinucleotide repeat expansion in the *HTT* gene located on the short arm of chromosome 4^1^. The disease is characterized by a triad of movement disorder, cognitive impairment, and behavioural disturbances.

There has been an increase in research lumbar punctures (LP) performed in people with HD. This is led by the current search for objective and quantitative biomarkers that could define disease onset, track its progression, and serve as outcome measures in clinical trials of disease-modifying therapeutics; and by the utilization of the intrathecal route of drug administration in several clinical development programs, most remarkably of the antisense oligonucleotides (ASOs).

LP is a generally well-tolerated procedure^2^, and experience from memory clinics and Parkinson’s disease shows that is a safe procedure, with relatively frequent mild and self-limiting adverse events and very rare severe or serious complications^3,4^.

However, the literature is limited when it comes to HD, where several factors may impact safety and feasibility in this population such as the relatively young age of participants usually recruited in HD clinical trials compared to those with dementia, Parkinson’s disease, and Alzheimer's disease; the characteristic brain atrophy associated with Huntington’s disease progression and its effect on the tolerance of post LP low-pressure syndrome; the variable degree of involuntary movement disorder that could pose some difficulties and challenges during the LP procedure; and also the neuropsychiatric manifestations that might affect their judgment, tolerability and their attitude toward the procedure.

A recent study by Rodrigues et al.^5^ addressed for the first time the safety profile of LP in a cohort of patients with Huntington's disease and controls who took part in the largest CSF collection initiative, HDClarity. They found that the adverse events rate in their cohort was similar to that reported in other neurological diseases or the general population and that age, gender, BMI, and disease burden score (DBS), as a surrogate of disease status, did not show association with the rate of post LP headache.

We adopted an individual patient data meta-analysis approach to build on this recently published work, by combining two cohorts to generate a substantially larger sample size (669 LP vs. 459 LP) and therefore higher statistical power; and also to increase the representation of premanifest HD, a population in which a higher risk of post-LP complications might be expected (given their young age); and finally to include substantially more controls, matched across the whole range.

This larger representation of subjects with HD in general, and of those at the premanifest stage in particular, are increasingly the focus of new therapeutic trials for HD, so we hoped that this approach would help to inform future clinical trials.

## Methods

### Study design

We conducted an individual patient data meta-analysis of two studies: HDClarity and HD-YAS.

The HDClarity study (NCT02855476)^5^ is an ongoing, international, multi-site, longitudinal CSF collection initiative aiming to recruit 1200 participants. The primary objective is to collect high-quality CSF samples for the evaluation of biomarkers and pathways that will enable the development of novel treatments for HD. The study protocol can be found at https://hdclarity.net.

The Huntington’s Disease Young Adult Study (HD-YAS) is a single centre cross-sectional study aimed at identifying the earliest signs of changes in brain structure and function caused by the HD mutation, including CSF biomarkers, in a cohort of 64 young adult premanifest gene carriers, selected to be relatively far from the onset, compared to 67 healthy controls^6^. The study protocol can be found at https://www.ucl.ac.uk/ion/sites/ion/files/hd-yas_protocol_v6.0_sep18_signed.pdf.

### Ethical considerations

The Declaration of Helsinki and the International Council for Harmonisation of Technical Requirements for Pharmaceuticals for Human Use (ICH) Good Clinical Practice (GCP) standards were followed. For HDClarity, all participating sites sought appropriate ethical approval in accordance with each country's specific legislation. HDClarity was approved by London—Camberwell St Giles Research Ethics Committee (16/LO/1878).

HD-YAS was approved by the Bloomsbury Research Ethics Committee (16/LO/1323). Participants had signed informed consent that is written and administered in compliance with the ICH-GCP requirement.

We confirm that we have read the Journal’s position on issues involved in ethical publication and affirm that this work is consistent with those guidelines.

### Study participants

We grouped participants into three categories: healthy controls, premanifest HD, and manifest HD.

Controls were defined as participants who were either at risk of inheriting the expanded mutant gene but have tested negative in predictive genetic testing (*HTT* gene CAG < 36) or were family members or friends who were not at risk of inheriting the expanded mutation, and therefore, they had not undergone predictive genetic testing.

HD gene expansion carriers (HDGEC) (*HTT* gene CAG ≥ 36) were grouped into either premanifest HD or manifest HD based on whether or not they had clinical diagnostic motor features of HD according to the Unified Huntington's Disease Rating Scale (UHDRS) Diagnostic Confidence Level (DCL) (i.e., manifest HD had UHDRS DCL = 4, while premanifest had DCL < 4).

We excluded from the analyses participants who did not undergo a LP.

### Study procedures

In HDClarity, LP procedures were performed at a sampling visit within 30 days of the screening visit. There was an optional repeat sampling visit 4 to 8 weeks after the initial visit. Some eligible participants were invited to participate in the study more than one time, at least 11 months after the previous screening visits. In HD-YAS, CSF collection was an optional study component and performed once only.

Both protocols mandated subjects to fast from midnight before the LP and the LP was performed by trained and experienced study site investigators. The HDClarity and HD-YAS protocols specified a 22G Whitacre (atraumatic) needle, and the method of CSF collection was via gravity dripping. The volume of the collected CSF was between 15 to 20 mL of CSF at each CSF sampling visit.

The number of lumbar puncture attempts that were permitted by the protocol was limited to 3 per visit. It was recommended to place the participants in the lateral decubitus position for CSF collection and to use the intervertebral space of L4/5 or L3/4 for CSF collection. According to the judgment of the site investigators, participants were advised to either lie flat for up to one hour after the lumbar puncture procedure or to mobilize immediately.

Upon discharge, participants were instructed by the study site staff to stay hydrated and take over-the-counter pain relief medication, and lie flat in the bed if they develop post LP syndrome. Participants were also instructed to avoid heavy lifting or strenuous exercise for 24 h post LP.

### Study assessments

The motor, cognitive and functional status was assessed using the UHDRS’99 from the core Enroll-HD battery^7^, including the total motor score (TMS), total functional capacity (TFC), independence scale (IS), symbol-digit modality test (SDMT), and stroop word reading (SWR). For HDClarity these were performed at either the screening visit before sampling or an associated Enroll-HD visit (https://www.enroll-hd.org) within the 2 months prior to screening. For HD-YAS they were performed on the day before sampling.

The composite Unified Huntington’s Disease Rating Scale (cUHDRS) was used as it is considered one of the most sensitive clinical measures for HD progression across the functional, motor, and cognitive domains^8–10^. We employed a calibrated iteration of the cUHDRS^11,12^.

$$cUHDRS=\frac{TFC-8.8}{2.8}-\frac{TMS-34.4}{17.4}+\frac{SDMT-25.2}{12.4}+\frac{SWR-58.0}{21.2}+10$$

The Disease burden score (DBS) was calculated for each HDGEC using the formula^13^:

$$DBS=\left(CAG-35.5\right)*age$$

### Monitoring and recording adverse events in each study

In both studies, study site staff were mandated by the protocol to call the participants 24 to 72 h after the sampling visit to record any adverse events. Participants were also instructed to contact the study site to report any adverse events at any time point post-procedure.

For the purpose of our analysis, all adverse events were included. Primary analyses focused on the occurrence of post-LP adverse events, headaches, and back pain.

### Data management

Included HDClarity sampling visits occurred from February 2016 to September 2019, while the HD-YAS visits occurred from August 2017 to March 2019. Only fully independently monitored visits with complete data which had undergone quality control were included: 60 out of 729 visits were excluded.

### Statistical methods

We performed an individual patient data meta-analysis to investigate the occurrence of post-LP adverse events, headaches, and back pain. All analyses were performed using Stata/SE 15.1 (StataCorp, College Station) software. The significance (alpha) level was defined as 0.05.

General demographic characteristics, clinical assessments, and lumbar puncture characteristics at the first sampling research visit for each participant were described and compared according to the participant’s disease status (control, premanifest and manifest) for the two studies together, without accounting for the study provenance. Continuous variables were reported as mean and SD; counts as median, IQR, minimum and maximum; and categorical variables as absolute (n) and relative frequencies (%). To investigate group differences, linear regression analysis was performed for dependent continuous variables, and logistic regression analysis was performed for dependent categorical variables.

Our outcomes of interest were the occurrence of post-LP adverse events, post-LP headaches, and post-LP back pain. Each of these outcomes was investigated independently.

Building a multivariable meta-analysis model to evaluate the odds of adverse events in each group of interest was an iterative process. The approach described below was repeated for each outcome of interest, first for all three studied participant subgroups and then for HDGEC only.

First, we explored several univariable generalized logistic models. All models had the outcome odds as the dependent variable and group membership as the fixed effect. To account for the research study's provenance (i.e., HDClarity or HD-YAS), we used a random intercept for the study. Given that a fraction of the participants from HDClarity (n = 170 [25.41%]) attended multiple visits, we tried to also include a random intercept for participants, nested into the study random effect to account for repeated measures. A negligible fraction (n = 2 [0.30%]) of participants provided data to both studies but we only included them once. We considered including a crossed random effect. Unfortunately, our data did not converge on models with crossed random effects or a nested participant random intercept. This was likely due to a lack of available information and/or variability across random effects. As such, we used a model with a random intercept for study only.

To ensure the internal validity of our final model structure we run several sensitivity analyses using less parsimonious models: a fixed effect logistic model only including the first sampling visit for each participant; a mixed-effect logistic model only including the first sampling visit for each participant and a random intercept for study; bootstrapped mixed-effect logistic model including one random sampling visit per participant.

Secondly, we selected which variables to include in the multivariable model. Age and CAG repeat count have a strong influence on HD-related phenomena^14–16^. We also included other covariates based on prior knowledge of their possible association with the development of adverse events: gender^3,4^ and Body Mass Index (BMI)^17,18^ for post-LP headaches. Also, the number of lumbar puncture attempts was included in the final multivariable model for post-LP back pain. As such, we decided to include them as fixed effects when possible. We investigated the associations of the following variables (age, gender, BMI, ethnicity, *HTT* CAG size, DBS, cUHDRS, TMS, TFC, IS, SDMT, SWRT, number of LP attempts, position, and space used during LP procedure and volume of CSF collected during the LP) with the exposure (group membership) and outcome of interest (adverse events or post-LP headaches or post-LP back pain) by means of univariable analyses. Variables considered associated with both the exposure and outcomes were included in the final model in a stepwise fashion, starting with the ones with a larger magnitude of effect and comparing sequential model performance based on the log-likelihood ratio test. Collinearity was evaluated with Spearman rank correlations and data spareness with cross-tabulation of data.

The final multivariable model was a multivariable logistic model with odds of the outcome of interest as the dependent variable, group membership, age, gender, CAG (HDGEC sensitivity analyses only), BMI (headaches model only) as fixed effects, and study provenance as a random intercept.

The final model was utilized to investigate the intergroup differences and to calculate the adjusted odds of each event for each studied group.

We also investigated potential effect modification by age and gender by including interactions of these with the group membership. In the interaction model, age was categorized according to the quantiles of distribution. To apply the interaction model for the outcome of back pain, we had to exclude the 4th quantile of age (57–77 years) due to data scarcity.

## Results

### Study participants and visit distribution

Data included were collected in the period between February 2016 and Sep 2019.

The final cohort included 497 participants (139 control, 179 premanifest HD, 179 manifest HD) from the two studies (395 participants from HDClarity and 102 participants from HD-YAS) with a median age of 43 years (range 19–77). The demographic and disease characteristics of the participants and the characteristics of the LP procedures are presented in Table 1. Participants at the premanifest stage were significantly younger (mean age was 37 years in premanifest versus 53 in the manifest group, *p* < 0.001). The number of male and female participants was well balanced (female 50.9%); however, this was not the case among subgroups where female participants made up 54.75% of the premanifest subgroup and 42.46% of the manifest subgroup. 97.8% of all participants were of Caucasian ethnicity. The mean BMI was 26.2, SD 5.02.

**Table 1:** Baseline demographics and disease characteristics and CSF sample methodology at first LP visit.

	All subjects	Healthy control	Premanifest	Manifest	Group membership *P*-value	HC vs PM *P*-value	PM vs M *P*-value
N	497	139	179	179			
Age (years)	43.94 (13.62)	41.92 (14.20)	36.693 (10.81)	52.76 (10.46)	< 0.001	< 0.001	< 0.001
Female	253 (50.91%)	79 (56.83%)	98 (54.75%)	76 (42.46%)	0.018	0.710	0.020
Caucasian	486 (97.79%)	136 (97.84%)	174 (97.21%)	176 (98.32%)	0.775	0.721	0.479
BMI	26.16 (5.02)	27.31 (5.56)	25.68 (4.97)	25.73 (4.47)	0.006	0.004	0.919
CAG	43.03 (2.53)	N/A	42.68 (2.26)	43.39 (2.73)	N/A	N/A	0.009
DBS	324.48 (113.98)	N/A	252.66 (79.81)	396.30 (96.49)	N/A	N/A	< 0.001
cUHDRS	15.54 (3.97)	18.05 (1.60)	17.65 (1.65)	11.33 (3.56)	< 0.001	0.154	< 0.001
UHDRS-TFC score	11.83 (2.41)	12.97 (0.21)	12.86 (0.62)	9.91 (3.16)	< 0.001	0.613	< 0.001
UHDRS-TMS	12.75 (19.54)	0.83 (1.82)	1.99 (3.27)	32.89 (20.45)	< 0.001	0.407	< 0.001
UHDRS-IS	93.68 (12.78)	99.96 (0.42)	99.55 (2.14)	82.93 (16.40)	< 0.001	0.715	< 0.001
SDMT	46.54 (17.29)	56.12 (11.69)	55.31 (11.20)	29.77 (13.23)	< 0.001	0.555	< 0.001
SWRT	86.83 (26.86)	103.27 (17.19)	98.42 (18.35)	62.19 (22.20)	< 0.001	0.285	< 0.001
Number of LP attempts	1.01 (1.60)	1.14 (0.39)	1.21 (0.49)	1.28 (0.56)	0.051	0.218	0.197
Lateral decubitus	428 (86.12%)	117 (84.17%)	159 (88.83%)	152 (84.92%)	0.419	0.226	0.275
LP site L3-L4 L4-L5 other	250 (50.40%) 213 (42.94%) 33 (6.65%)	80 (57.55%) 52 (37.41%) 7 (5.04%)	86 (48.31%) 80 (44.94%) 12 (6.74%)	84 (46.93%) 81 (45.25%) 14 (7.82%)	0.196–0.871	0.129–0.831	0.870–0.738
CSF volume	18.96 (3.55)	19.57 (3.77)	19.21 (2.44)	18.23 (4.15)	0.002	0.363	0.009
CSF RBCs count	25.15 (171.92)	9.66 (30.72)	16.21 (94.32)	46.83 (271.04)	0.114	0.737	0.096

Continuous variables are reported as mean (standard deviations). Categorical variables are reported as absolute and relative frequencies.

*BMI* body mass index*, CAG* HTT CAG size*, DBS* Disease Burden Score*, cUHDRS* composite Unified Huntington’s Disease Rating Scale*, TMS* UHDRS Total Motor Score*, TFC* UHDRS Total Functional Capacity*, IS* UHDRS Independence Scale*, SWR* Stroop Word Reading test*, SDMT* Symbol Digits Modality Test*, RBCs* Red Blood Cells.

The total number of included LPs was 669 (104 LPs from the HD YAS and 565 LPs from the HDClarity). Within HDClarity, 395 LPs were done at the first sampling visit, 156 LPs were done at a second sampling visit and 14 LPs were done at a 3rd sampling visit.

Out of 669 LP procedures, 492 LP procedures were done in patients who were either premanifest (231 LP procedures) or manifest (261 LP procedures).

Overall, 342 participants had a single LP and 155 had 2 or more LPs.

The majority of the lumbar puncture procedures were done in the lateral decubitus position (86%) via the L3-L4 (50%) space. 550 LP procedures (82%) were successful on the first attempt. The mean CSF volume collected was 19 ml (SD 3.55). 28.7% of all the CSF samples had erythrocyte counts of more than 5 cells/uL. 18.7% contained more than 10 cells/uL.

### Univariable analyses

#### Adverse event description

Overall, there were 184 (27.5%) LP visits where one or more adverse event was reported (Table 2). The two most common adverse events were: post LP headache and post LP back pain, accounting for 116 (17.3%) and 68 (10.2%) of all lumbar puncture visits, respectively.

**Table 2:** Observed frequencies of adverse events at all included LP visits.

All procedures	All procedures 669 (100%)	LP in Control 177 (100%)	LP in Premanifest 231 (100%)	LP in Manifest 261 (100%)
LP with any adverse event	(184) 27.5%	51 (28.81%)	84 (36.36%)	49 (18.77%)
LP with Post LP headache	(116) 17.34%	36 (20.34%)	53 (22.94%)	27 (10.34%)
LP with Post LP back pain	(68) 10.16%	14 (7.91%)	35 (15.15%)	19 (7.28%)

Most headache events resolved spontaneously, with or without over-the-counter analgesics, and were classed as mild to moderate except in one case which was graded as severe. Epidural blood patch was required in only 1 case.

All back pain events were graded between mild and moderate and resolved without sequela, with or without simple analgesics.

Of all lumbar puncture visits, 2.54% were associated with other events not classified as headache or back pain, for example, puncture site swelling, bruising or hematoma at the site of the procedure, nausea or vomiting, and vasovagal syncope.

Among the HDGEC group, the percentage of LP with adverse events was 27.03%, while the percentage in the control group was 28.81%.

Table 3 illustrates the baseline characteristics in relation to the development of adverse events.

**Table 3:** Baseline demographics and CSF sample methodology in relation to the development of adverse events.

Adverse events	Total	Procedures without adverse events (100%)	Procedures with Adverse events (100%)	*p*-value
	N = 497	N = 349	N = 148	
Age	44 (14)	46 (13)	40 (13)	< 0.001
Female	253 (51%)	168 (48%)	85 (57%)	0.058
Caucasian	486 (98%)	343 (98%)	143 (97%)	0.25
BMI	26.16 (5.02)	26.26 (5.10)	25.91 (4.83)	0.47
**HD category**				
Control	139 (28%)	98 (28%)	41 (28%)	< 0.001
Premanifest	179 (36%)	109 (31%)	70 (47%)	
Manifest	179 (36%)	142 (41%)	37 (25%)	
DBS	324.48 (113.98)	340.69 (113.27)	286.34 (106.79)	< 0.001
UHDRS-TFC score	11.83 (2.41)	11.61 (2.61)	12.34 (1.71)	0.002
UHDRS- IS	93.68 (12.78)	92.61 (13.83)	96.22 (9.45)	0.004
UHDRS- TMS	12.75 (19.54)	15.04 (21.24)	7.37 (13.39)	< 0.001
SWRT	86.83 (26.86)	84.80 (27.81)	91.57 (23.93)	0.010
SDMT	46.54 (17.29)	44.67 (17.66)	50.87 (15.60)	< 0.001
cUHDRS	15.54 (3.97)	15.14 (4.17)	16.47 (3.31)	< 0.001
Number of LP attempts	1 (0)	1 (0)	1 (1)	0.71
**LP site**				
L4/5	213 (43%)	152 (44%)	61 (41%)	0.45
L3/4	250 (50%)	176 (50%)	74 (50%)	
other	33 (7%)	20 (6%)	13 (9%)	
Lateral decubitus	428 (86%)	299 (86%)	129 (87%)	0.66
CSF volume	19 (4)	19 (3)	19 (4)	0.90
CSF RBCs count	25 (172)	32 (204)	9 (31)	0.17

Continuous variables are reported as mean (standard deviations). Categorical variables are reported as absolute and relative frequencies.

*BMI* body mass index, *CAG* HTT CAG size, *DBS* Disease Burden Score, *cUHDRS* composite Unified Huntington’s Disease Rating Scale, *TMS* UHDRS Total Motor Score, *TFC* UHDRS Total Functional Capacity, *IS* UHDRS Independence Scale, *SWR* Stroop Word Reading test, *SDMT* Symbol Digits Modality Test, *RBCs* Red Blood Cells.

The unadjusted odds ratio of developing adverse events was lower in the manifest subgroup compared to the control subgroup (Table 4); however, the results were non-significant (OR 0.66, 95%CI 0.41–1.07, *p* = 0.094). The premanifest subgroup compared to control had an odds ratio of 1.45, 95% CI 0.95–2.22, *p* = 0.88.

**Table 4:** Univariable analysis of all adverse events, headache and back pain.

Univariable analysis	All adverse events		Headache		Back pain	
	Odd ratio	*P*-value	Odd ratio	*P*-value	Odd ratio	*P*-value
Age	0.958	< 0.001	0.971	< 0.001	0.953	< 0.001
BMI	0.989	0.541	0.987	0.540	0.990	0.694
Sex	1.658	0.004	1.614	0.022	1.360	0.225
CAG	1.054	0.207	1.027	0.603	0.991	0.876
DBS	0.997	0.003	0.997	0.044	0.995	0.001
cUHDRS	1.108	< 0.001	1.138	< 0.001	1.069	0.051
Pre-M vs Control 95% CI	1.448 [0.95–2.22]	0.088	1.184 [0.73–1.91]	0.491	2.079 [1.08–3.99]	0.028
M vs Control 95% CI	0.662 [0.41–1.07]	0.094	0.501 [0.28–0.89]	0.019	0.914 [0.45–1.87]	0.806
HDGEC vs Control 95% CI	1.059 [0.71–1.58]	0.779	0.862 [0.55–1.36]	0.523	1.435 [0.78–2.65]	0.249
TFC score	1.182	0.001	1.214	0.003	1.138	0.056
TMS	0.973	< 0.001	0.968	< 0.001	0.972	0.004
UHDRS-IS	1.031	0.001	1.041	0.001	1.033	0.019
SDMT	1.025	< 0.001	1.027	< 0.001	1.014	0.061
SWRT	1.011	0.002	1.014	0.001	1.005	0.308
Number of LP attempts	1.158	0.395	1.092	0.669	1.084	0.746
LD position	1.047	0.855	1.111	0.729	1.110	0.782
Site L3-L4 vs L4-L5 Others vs L4-L5	1.029 2.145	0.881 0.017	0.945 1.972	0.800 0.062	1.191 1.905	0.526 0.142
CSF volume	0.985	0.522	1.026	0.412	1.025	0.522
CSF RBCs count	0.999	0.368	0.999	0.504	0.995	0.356

*BMI* body mass index, *CAG* HTT CAG size, *DBS* Disease Burden Score, *cUHDRS* composite Unified Huntington’s Disease Rating Scale, *Pre-M* Premanifest HD, *M* Manifest, *HDGEC* Huntington's disease gene expansion carrier, *TMS* UHDRS Total Motor Score, *TFC* UHDRS Total Functional Capacity, *IS* Independence Scale, *SWR* Stroop Word Reading test, *SDMT* Symbol Digits Modality Test, *LD* Lateral Decubitus, *RBCs* Red Blood Cells.

The unadjusted odds ratio of developing headache was significantly lower in the manifest subgroup compared to the controls (OR 0.50, 95% CI 0.28–0.89, *p* = 0.019). However, since the control group was matched to the overall HDGEC population, the manifest subgroup was older than the control group on average (mean age was 52.76 in manifest versus 43.94 in healthy control). There was no difference in the odds ratio of developing post LP back pain between the two groups (OR 0.91, 95% CI 0.45–1.87, *p* = 0.81).

Age and gender were not found to be effect modifiers for any of the studied outcomes (adverse events, headache, and back pain) and the disease category.

#### Factors affecting the incidence of post-LP adverse events

The mean age for all participants who developed adverse events was lower (mean 40, SD 13) compared to those who did not develop adverse events (mean 46, SD 13, *p* < 0.001). The OR for adverse events for each yearly increment in age was 0.96. This age difference was also significant in HDGEC.

The mean cUHDRS was higher in participants with adverse events (mean 16.47, SD 3.31) than in participants without adverse events (mean 15.14, SD 4.17, *p* < 0.001).

The mean BMI was similar (mean 26, SD 5) among those who developed any adverse events, headache, or back pain, and those who did not, with an odds ratio around 1 (OR = 0.99) and with a non-significant *p*-value (*p* = 0.54 for adverse events and headache, *p* = 0.69 for back pain).

The LP characteristics distribution was similar among participants who experienced and who did not experience adverse events.

#### Factors affecting the incidence of post-LP headaches

The manifest group of participants had the least number of headache events (22%) compared to the control and premanifest subgroups (30% and 48% respectively) (Table 5).

**Table 5:** Baseline demographics and CSF sample methodology in relation to the development of headache post LP.

Headache	Total	Procedures without adverse events (100%)	Procedures with Adverse events (100%)	*p*-value
	N = 497	N = 404	N = 93	
Age	44 (14)	45 (14)	41 (13)	0.007
Female	253 (51%)	198 (49%)	55 (59%)	0.078
Caucasian	486 (98%)	396 (98%)	90 (97%)	0.46
BMI	26.16 (5.02)	26.21 (5.04)	25.91 (4.95)	0.61
**HD category**				
Control	139 (28%)	111 (27%)	28 (30%)	0.003
Premanifest	179 (36%)	134 (33%)	45 (48%)	
Manifest	182 (36%)	159 (39%)	20 (22%)	
DBS	324.48 (113.98)	334.32 (113.63)	280.17 (105.46)	< 0.001
UHDRS-TFC score	11.83 (2.41)	11.68 (2.58)	12.46 (1.28)	0.005
UHDRS- IS	93.68 (12.78)	92.83 (13.70)	97.15 (6.61)	0.004
UHDRS- TMS	12.75 (19.54)	14.25 (20.72)	6.29 (11.24)	< 0.001
SWRT	86.83 (26.86)	85.07 (27.32)	94.41 (23.17)	0.002
SDMT	46.54 (17.29)	45.29 (17.41)	51.89 (15.74)	< 0.001
cUHDRS	15.54 (3.97)	15.25 (4.11)	16.79 (3.03)	< 0.001
Number of LP attempts	1 (0)	1 (0)	1 (1)	0.85
**LP Site**				
L4/5	213 (43%)	173 (43%)	40 (43%)	0.93
L3/4	250 (50%)	204 (50%)	46 (49%)	
Other	33 (7%)	26 (6%)	7 (8%)	
Lateral decubitus	428 (86%)	347 (86%)	81 (87%)	0.76
CSF volume	19 (4)	19 (4)	19 (2)	0.12
CSF RBCs count	25 (172)	30 (190)	6 (17)	0.24

Continuous variables are reported as mean (standard deviations). Categorical variables are reported as absolute and relative frequencies.

*BMI* body mass index, *CAG* HTT CAG size, *DBS* Disease Burden Score, *cUHDRS* composite Unified Huntington’s Disease Rating Scale, *TMS* UHDRS Total Motor Score, *TFC* UHDRS Total Functional Capacity, *IS* UHDRS Independence Scale, *SWR* Stroop Word Reading test, *SDMT* Symbol Digits Modality Test, *RBCs* Red Blood Cells.

The participants who developed headaches post LP were younger compared to those who did not. (mean 41, SD 13 and mean 45, SD 14) (*p* = 0.007).

This age difference was also significant among the subgroup of gene expansion carriers (manifest and premanifest).

Females had more headache events post LP than males (59%); however, this difference was not significant (*p* = 0.078), and the same was observed in the gene expansion carrier group of participants (57% of those who developed headaches were females, *p* = 0.14).

There was no significant difference in the BMI of the participants with and without headache (mean BMI = 25.91 compared to 26.21), *p* = 0.61.

#### Factors affecting the incidence of post-LP back pain

The mean age for participants who developed back pain following LP was lower when compared to participants who did not have back pain post LP (mean 37, SD 12 and mean 45, SD 14, *p* < 0.001) (Table 6).

**Table 6:** Baseline demographics and CSF sample methodology in relation to the development of back pain post LP.

Back pain	Total	Procedures without adverse events (100%)	Procedures with Adverse events (100%)	*p*-value
	N = 497	N = 441	N = 56	
Age	44 (14)	45 (14)	37 (12)	< 0.001
Female	253 (51%)	222 (50%)	31 (55%)	0.48
Caucasian	486 (98%)	433 (98%)	53 (95%)	0.090
BMI	26.18 (5.02)	26.20 (5.07)	25.79 (4.64)	0.56
**HD category**				
Premanifest	139 (28%)	127 (29%)	12 (21%)	0.033
Manifest	179 (36%)	150 (34%)	29 (52%)	
Control	182 (36%)	167 (37%)	15 (27%)	
DBS	324.48 (113.98)	331.16 (112.78)	276.45 (112.24)	0.003
UHDRS-TFC score	11.83 (2.41)	11.78 (2.48)	12.25 (1.67)	0.17
UHDRS- IS	93.68 (12.78)	93.29 (13.29)	96.79 (7.10)	0.054
UHDRS- TMS	12.75 (19.54)	13.52 (20.25)	6.73 (11.00)	0.014
SWRT	86.83 (26.86)	86.52 (27.34)	89.21 (22.81)	0.48
SDMT	46.54 (17.29)	46.08 (17.64)	50.11 (13.89)	0.01
cUHDRS	15.54 (3.97)	15.44 (4.08)	16.30 (2.97)	0.13
Number of LP attempts	1 (0)	1 (0)	1 (1)	0.81
**LP Site**				
L4/5	213 (43%)	191 (43%)	22 (39%)	0.17
L3/4	250 (50%)	223 (51%)	27 (48%)	
Other	33 (7%)	26 (6%)	7 (13%)	
Lateral decubitus	L3/4	378 (86%)	50 (89%)	0.47
CSF volume	Other	19 (3)	19 (5)	0.31
CSF RBCs count	25 (172)	27 (182)	7 (15)	0.40

Continuous variables are reported as mean (standard deviations). Categorical variables are reported as absolute and relative frequencies.

*SD* Standard deviation, *BMI* body mass index, *CAG* HTT CAG size, *DBS* Disease Burden Score, *cUHDRS* composite Unified Huntington’s Disease Rating Scale, *TMS* UHDRS Total Motor Score, *TFC* UHDRS Total Functional Capacity, *IS* UHDRS Independence Scale, *SWR* Stroop Word Reading test, *SDMT* Symbol Digits Modality Test, *RBCs* Red Blood Cells.

The female percentage in the group who developed back pain after LP was also higher than those who did not develop back pain (55% compared to 50%) with a non-significant p-value (*p* = 0.48).

The BMI mean was also not significantly different between the two groups (mean = 25.79 in the group who developed back pain compared to mean = 26.20 in the other group) (*p* = 0.56).

### Multivariable analysis

The results of the final multivariable analysis models showed that the adjusted odds ratio (OR) for developing adverse events in premanifest compared to control was 1.18 (95% CI 0.76–1.84), *p*-value 0.451.

The adjusted odds ratio for manifest compared to control was 0.87, 95% CI 0.53–1.42, *p*-value 0.578.

While for developing headaches, the adjusted odds ratio (OR) was 1.06 in premanifest versus control, (95% CI 0.65–1.73), *p*-value 0.82 and it was 0.55, 95% CI 0.31–0.98, *p*-value 0.44 in manifest compared to control.

The adjusted odds ratio (OR) for developing backache in premanifest versus control was 1.70 (95% CI 0.87–3.32), *p*-value 0.121.

**Figure1:**
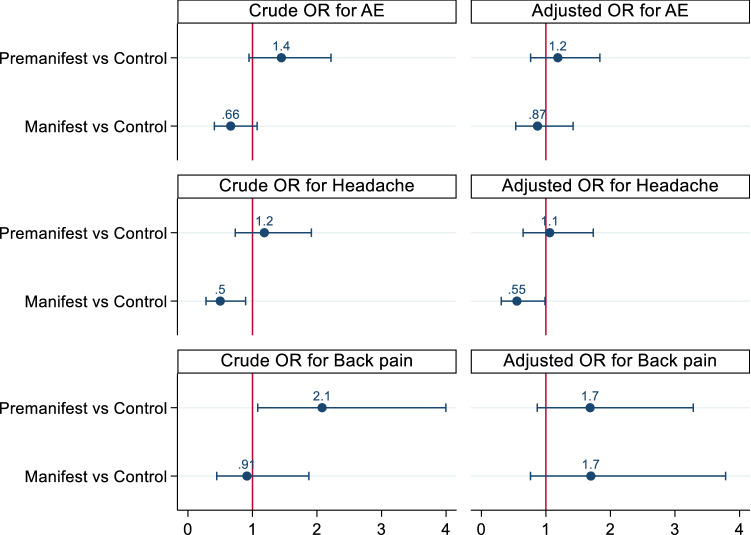
Odds ratio and 95%CI of adverse events in premanifest and manifest participants when compared to control participants. *OR* odds ratio.

The adjusted odds ratio for developing adverse events in manifest versus control was 1.69, and the 95% Conf. Interval [0.76–3.77], *p*-value 0.202 (Table 7), Fig. 1.

**Table 7:** Results of multivariable analysis of models in premanifest compared to control participants and in manifest compared to control participants.

Multivariable analysis	Adverse events	Headache	Back pain
	N = 657	N = 657	N = 657
Premanifest vs control OR 95% CI	1.18 [0.76–1.84]	1.06 [0.65–1.73]	1.70 [0.87–3.32]
Manifest vs control OR 95% CI	0.87 [0.53–1.42]	0.55 [0.31–0.98]	1.69 [0.76–3.77]
HDGEC vs control OR 95% CI	1.046 [0.70–1.56]	0.832 [0.54–1.30]	1.70 [0.91–3.20]

*HDGEC* HD gene expansion carrier, *OR* Odd ratio.

## Discussion

Here we performed the largest investigation to date of LP procedure safety and feasibility in patients with Huntington’s disease, using a much larger sample size than prior reports, to improve the statistical power and including a larger number of premanifest participants towards whom new treatment trials will be directed, analysing data from 669 LP visits over more than 3 years. We have paid special attention to studying disease status (control, manifest or premanifest) as a potential factor that was not previously addressed in other studies.

The incidence of adverse events, post LP headache, and post LP back pain among our HDGEC population in this analysis is aligned with that reported in other studies in Huntington’s disease^19^ or other populations such as Parkinson’s disease^3^, Alzheimer’s Disease^20^, and in a large study of patients attending memory clinics^4^.

Adverse events were generally significantly higher in younger participants. The same is also true for post lumbar puncture headache and post lumbar puncture back pain. These results confirm what is previously known about older age as a preventive factor^21^,^22^.

In alignment with the above, the rate of post LP headache in HD-YAS participants, in whom a mean age of 29.16 years, was much higher (29.81%) than the rate of post LP headache among HDClarity participants (15.04%, who have a mean age of 49.38 years).

The impact of age on adverse events rate may also partially explain the results of disease status in this analysis. As the initial univariable analysis showed that the manifest subgroup of patients had fewer adverse events when compared to premanifest and control subgroups, this was not the case after adjusting for covariates like age and gender.

However, the final multivariable analysis model confirmed that the disease status did not influence the rate of developing adverse events.

The reduced incidence of post LP headache in older age groups was hypothetically linked in several studies to brain atrophy^23–25^; thus, studying the association between brain volume and the development of post LP headache in patients with Huntington’s disease, independent of age, would be an interesting point to be tested in future studies.

Consistent with other studies^3,26^, female participants were found to be at higher risk of developing any adverse events, headaches, and back pain than male participants. This was also true in the sub-analysis of CAG gene expansion carrier participants. There is no clear explanation for the gender effect.

In our analysis, we had a wide range of BMI values ranging from 16 to 55 kg/m2 as neither of the included studies in this analysis had any specific inclusion criteria related to participants’ weight or BMI. BMI was not found to affect the incidence of post-LP adverse events. This is consistent with previous studies^3^.

Despite the relatively high volume of CSF collected (20 ml) during lumbar puncture procedures included in our analysis, this did not result in a rise in the rate of adverse events compared to other studies with lower CSF volume collected and provides reassurance that collecting such volumes is safe and well-tolerated.

The position of the participant during the lumbar puncture procedure did not appear to have any significant impact on the incidence of adverse events. Hence, we would recommend that future designs of clinical trials allow the site investigators to choose the appropriate position for their participants based on their experience and preference unless either of the positions is preferred for a different reason e.g., an upright sitting position to measure the CSF opening pressure.

A major strength of this work is that it pools participants from two studies to improve statistical power and clinical diversity; however, this also comes at the cost of potential variability between studies. The mean age for those who came from HD-YAS was much younger than those from HDClarity, also, while both studies had premanifest and control subjects, the manifest group only came from the HDClarity study. To overcome this, the multivariable model was built accounting for study provenance.

HDClarity is a large multisite study and data included in this analysis came from different sites globally which subsequently could be a cause of variation in reporting and monitoring adverse events.

The effect of large CSF volume withdrawn and the position of the participant during the LP procedure should be interpreted with caution as both source studies were mandated by the protocol to collect the same volume of CSF (20 ml) and to favour the same position for all participants (lateral decubitus).

Needle gauge is well-established to be significantly associated with the rate of development of post LP Headache^2,3,25^. In our study, this factor could not be analysed in relation to the development of adverse events as only one size (22G) was used in all participants; however, the reported rate of post LP headache in our study, in which 22G needle size was used, is generally aligned with the reported rate of post LP headache in studies where needle size 24G was used such as in the Tominersen ASO development program^19,27^.

Given that many potential factors could possibly affect the incidence rate of post LP adverse events, a larger sample size would be more appropriate and will give better statistical power.

It is important to note that our analysis did not take into account the duration of events of interest and potential right-censoring related to the limited observation period after the visits.

Another limitation in our analysis is the inclusion of all reported headache events. There were three cases of tension headaches and four cases of migraine headaches that were described by the study site investigator as unrelated to LP procedure, there were 17 events of non-postural mild headache related to the LP but not classified as a typical low-pressure syndrome.

An important point to reflect on, is that our cohort in this analysis came from subjects selected for participation in observational research; their characteristics may not be representative of people with HD in general, or of participants in interventional therapeutic trials.

In summary, our results suggest that the lumbar puncture procedure is safe and tolerable in premanifest and manifest Huntington's disease subjects. This information is useful to be communicated to the Huntington’s disease community to encourage participation in clinical trials and to reduce any anxiety relating to the procedure.

## Supplementary Information

Supplementary Information.

## Data Availability

All data generated during this study are not publicly available, however, they are available from the corresponding author on reasonable request and with permission of CHDI Foundation, Inc.
