# Supplementary material for: Lumbar puncture safety and tolerability in premanifest and manifest Huntington’s disease: a multi-analysis cross-sectional study
Source: Sci Rep. 2022 Nov 1;12:18377. doi: 10.1038/s41598-022-21934-6 (PMC9626630; doi:10.1038/s41598-022-21934-6)
Supplement: Supplementary file 1 — Supplementary Information. [file 41598_2022_21934_MOESM1_ESM.docx]

Supplementary Material

**Lumbar puncture safety and tolerability in premanifest and manifest Huntington’s disease: a multi-analysis cross-sectional study**

**Supplementary Material 1. HDClarity Investigators**

**Central Coordination**

University College London: Edward J Wild (Chief Investigator), Gail Owen (Study Manager),

Filipe B Rodrigues (Quality Control Officer), Katarzyna Schubert (Study Coordinator), Seema

Maru (Study Coordinator), Alexander Lowe (Research Assistant), Stefanie Gosling (former

Study Coordinator).

CHDI Foundation: Robi Blumenstein (President), Cristina Sampaio (Chief Clinical Officer),

Eileen Neacy (Chief Operating Officer), Swati Sathe (Medical Director, Clinical Research),

Anka G Ehrhardt (Director, Bio Fluid Clinical Research), Elena Pak (Clinical Research Program

Manager and Study Lead), Shilpa Deshpande (Director, Clinical Operations), Sherry Lifer

(Director, Contract Finance & Operations), Julia Keklak (Clinical Program Manager,

Biorepository), Dipinder Kaur (former Clinical Biorepository Program Manager), Jamie Levey

(Co-Director, Clinical Research Platform), Olivia Handley (Enroll-HD Global Project Manager),

Jenny Townhill (Enroll-HD Trial Manager), Mette Gilling (Enroll-HD Scientific Project

Manager).

**Study Sites (February 2016 to September 2019)**

*Center Movement Disorders, CA:*

Mark Guttman (Principal Investigator), Bhavpreet Dam (Sub-Investigator), Ragani Srinivasan

(Sub-Investigator), Ben Safa (Sub-Investigator), Keith Tanner (Sub-Investigator), Fahad Alam

(Sub-Investigator), Jonielyn Carlos (Study Coordinator), Teena Kailasanathan (Study

Coordinator), Marijana Pajic (Study Coordinator), Theresa Moore (Study Coordinator), Susan

Whyte (Study Coordinator), Tania Mani (Study Coordinator), Marie Villagonzalo (Study

Coordinator), Kim Thompson (Study Coordinator).

*University British Columbia, CA:*

Blair Roland Leavitt (Principal Investigator), Lynn Alison Raymond (Sub-Investigator), Mike

Adurogbangba (Study Coordinator), Fabricio J Pio (Rater) Emma Peachey (Research Assistant),

Jonathan Squires (Sub-Investigator), Valerie O’Neill (Study Coordinator), Tuan Le (Study

Coordinator), Rachel Wan (Research Assistant), Devine Calanog (Research Assistant) and Tariq

Aziz (Lab Manager).

*George Huntington Institute, DE:*

Ralf Reilmann (Principal Investigator), Stefan Bohlen (Deputy / Sub-Investigator), Anabel

Ruesenberg (Sub-Investigator), Anja Kletsch (Study Coordinator/Rater), Laura Spital (Study

Coordinator/Rater), Paula Raulet (Study Coordinator/Rater).

*St Josef And Elisabeth Hospital, DE:*

Carsten Saft (Principal Investigator), Sarah Maria von Hein (Sub-Investigator), Jannis

Achenbach (Sub-Investigator), Barbara Kaminski (Study Coordinator/Study Nurse), Daniela

Kaminski (Study Nurse).

*University Hospital Erlangen, DE:*

Jürgen Winkler (Representative Principal Investigator 07-MAR-2018 until 14-JAN-2019;

Principal Investigator since 15-JAN-2019), Zacharias Kohl (Principal Investigator 07-Mar-2018

until 14-JAN-2019), Franz Marxreiter (Sub-Investigator 07-MAR-2018 until 14-JAN-2019;

Representative Principal Investigator since 15-JAN-2019), Martin Regensburger (SubInvestigator), Susanne Seifert (Study Coordinator), Holger Meixner (Laboratory Technical

Staff), Jasmin Burczyk (Study Administration), Pia-Marie Pryssok (Study Nurse).

*University Hospital Ulm, DE:*

Jan Lewerenz (Principal Investigator), Bernhard Landwehrmeyer (Representative Principal

Investigator), Katrin Lindenberg (Sub-Investigator), Alzbeta Mühlbeck (Sub-investigator), Hela

Jerbi (Study Nurse), Moreen Igbineweka (Study Nurse), Sonja Trautmann (Study Nurse), Ariane

Schneider (Study Nurse), Heidi Jäger (Laboratory Technical Staff).

*BirmSolNHSFounTrust, GB:*

Hugh Rickards (Principal Investigator), Diana Crossley (Sub-Principal Investigator), Aaron

Sturrock (Sub-Principal Investigator), Jennifer De Souza (Study Coordinator/Rater), Theresa

Brady (Research Nurse), Anna Finnegan (Research Nurse), Samantha Timmis (Research Nurse),

Maria Bandeira (Data Officer), Tracy Soulsby (Data Officer), Nula Kelly (Research Nurse),

Melissa Wardale (Lab Manager), Fahd Niaz (Data Officer).

*GreatGlasgowHealthBoard, GB:*

Stuart Ritchie (Principal Investigator), Stuart Affleck (Sub-Investigator), Paul Gallagher (SubInvestigator), Stephanie Cowan (Sub-Investigator), Sarah Martin (Sub-Investigator), Shoshana

Cross (Sub-Investigator), Gillian Scott (Sub-Investigator), Craig Patrick (Sub-Investigator),

Catherine Deith (Study Coordinator), Carol Malcolmson (Study Coordinator), Murray

Sutherland (Research Nurse), Scott Farmer (Research Nurse), Lanah Dunsmuir (Research

Nurse), Anne Lewis (Lab Manager).

*LeedsTeachHospTrust, GB:*

Jeremy Cosgrove (Principal Investigator), Callum Schofield (Study Coordinator), Alan Liu

(Research Nurse), Helena Baker (Biomedical Scientist), Jodie Sedgwick (Biomedical Scientist).

*StGeorgeHealthTrust, GB:*

Nayana Lahiri (Principal Investigator), Bhavini Patel (Sub-Investigator), Sally Goff (Study

Coordinator), Uruj Anjum (Study Coordinator), Chandni Patel (Study Coordinator).

University Cambridge, GB:

Roger Barker (Principal Investigator), Thomas Stoker (Sub-Investigator), Katie Andresen (Study

Coordinator/Rater).

*University College London, GB:*

Edward J Wild (Chief Investigator/Principal Investigator), Filipe Brogueira Rodrigues (SubInvestigator), Lauren M Byrne (Study Coordinator/Rater), Rosanna Tortelli (Sub-Investigator),

Peter McColgan (Sub-Investigator), Mike Flower (Sub-Investigator), Carlos Estevez-Fraga (SubInvestigator), Paul Zeun (Sub-Investigator), Carolin Koriath (Sub-Investigator), Edwina

Saunders (Research Nurse), Mila Resuello-Dauti (Research Nurse), Laura Hennelly (Research

Nurse), Nuria Mora Morell (Research Nurse), Mark Elliot (Nurse Assistant), Rhoda Castaneda

(Study Coordinator), Martha S. Foiani (Research Technician), Jamie Toombs (Research

Technician), Elena Veleva (Research Technician), Michael Chou (Research Technician).

*JohnsHopkinsUniv, US:*

Jee Bang (Principal Investigator), Christopher Ross (Sub-Investigator), Kia E Ultz (Study

Coordinator/Rater), Jacqueline V Bran (Study Coordinator), Eka Chighladze (Lab Technician),

Priyanka Rauniyar (Lab Technician), Chelsy Eddings (Lab Technician).

*WakeForestUniv, US:*

Francis Walker (Principal Investigator), Clarisse Goas (Sub-Investigator), Victoria Hunt

(Research Nurse), Christine O’Neill (Study Coordinator/Rater), Jessica Bargoil (Study

Coordinator/Rater), Sara Byerly (CRU Manager), Cathy Gilkey (Lab Technician), LuAnn

Mascorro (Lab Technician).

*UnivTexasHlthCntrHous, US:*

Erin Furr-Stimming (Principal Investigator), David Hunter (Sub-Investigator), Beth Latham

(Study Coordinator/Rater), Jamie Sims (Study Coordinator/Rater), Brittany Duncan (Study

Coordinator/Rater).

**Supplementary Material 2. HD-YAS Investigators**

K Osborne-Crowley, C Parker, J Lowe, C Estevez-Fraga, K Fayer, H Wellington, FB Rodrigues, LM Byrne, A Heselgrave, H Hyare, H Zetterberg, EJ Wild, H Zhang (University College London), C O’Callaghan, Christelle Langley, TW Robbins, BJ Sahakian (University of Cambridge), C Sampaio (CHDI Management/CHDI Foundation Inc), D Langbehn (University of Iowa).
